# Designing of high entropy alloys with high hardness: a metaheuristic approach

**DOI:** 10.1038/s41598-024-57094-y

**Published:** 2024-04-02

**Authors:** Ansh Poonia, Modalavalasa Kishor, Kameswari Prasada Rao Ayyagari

**Affiliations:** BML Munjal University, Gurugram, Haryana 122413 India

**Keywords:** Composition optimization, Phase prediction, High entropy alloys, Differential evolution, Hardness prediction, Materials science, Mathematics and computing

## Abstract

The near-infinite compositional space of high-entropy-alloys (HEAs) is a huge resource-intensive task for developing exceptional materials. In the present study, an algorithmic framework has been developed to optimize the composition of an alloy with chosen set of elements, aiming to maximize the hardness of the former. The influence of phase on hardness prediction of HEAs was thoroughly examined. This study aims to establish generalized prediction models that aren’t confined by any specific set of elements. We trained the HEA identification model to classify HEAs from non-HEAs, the multi-labeled phase classification model to predict phases of HEAs also considering the processing route involved in the synthesis of the alloy, and the hardness prediction model for predicting hardness and optimizing the composition of the given alloy. The purposed algorithmic framework uses twenty-nine alloy descriptors to compute the composition that demonstrates maximum hardness for the given set of elements along with its phase(s) and a label stating whether it is classified as HEA or not.

## Introduction

HEAs are a new set of materials that surpass conventional metallic alloys with extraordinary mechanical properties^1^. Since their introduction in 2004, these materials have been developed and adapted as key focus areas due to their vast compositional space compared to conventional alloys. Later, HEAs were referred to as complex concentrated alloys, multi-complex alloys, multi-principal alloys, and compositionally complex alloys. The applications of HEAs in aerospace, marine, cryogenic, space, and transportation have increased over the past 2 decades due to their exceptional properties such as high strength with ductility, high hardness, toughness, high-temperature resistance, thermal stability, good wear resistance, and magnetic properties^2,3^. Multiple investigations are underway for the development of HEAs such as refractory alloys, transition metal HEAs, and HE-bulk metallic alloys. Even though studies on HEAs are increasing at a rapid pace, the infinite possibilities of elemental compositional space led to time and resource consumption. A little variation in the composition of multi-principal elements results in different properties. To overcome this problem, the development of computational techniques to optimize the composition of HEAs and decrease the number of experiments is necessary. Methods such as density functional theory (DFT), empirical relations, calculations of phase diagrams (CALPAD), and molecular dynamics provide solutions to phase predictions and composition optimization. However, their predictions are limited to a given alloy system at a given time making them resource and time-intensive. This has led to the development of machine learning (ML) methods with higher prediction accuracy to come in play. Over the past few years, phase predictions and optimization of descriptors have been developed in parallel, using ML researchers have probed deeply into the interrelationship between phases and key thermodynamic and physical descriptors to improve prediction accuracy^4–9^. Yan et al.^10^ developed nine different classification models with 70 appropriate descriptors using a genetic algorithm (GA). Their study provided a comprehensive framework for descriptor subset selection in phase prediction. Zhang et al.^11^ model considers four relevant descriptors, including H_e_ (elastic energy of the alloy), H_l_ (liquid phase mixing enthalpy), ΔS_mix_ (mixing entropy of the alloy), and atomic size difference (Δ), which were found to have a significant impact on phase prediction. By taking these descriptors into account, the model was able to accurately predict the phase behavior of HEAs, which is important for developing new materials with desirable properties. Jain et al.^12^ developed artificial neural network (ANN) models to predict the microhardness of cast FeCoNiCrMnVNb_x_ eutectic HEAs. The developed algorithms were found to be generic and applicable for predicting other mechanical properties as well but limited to a particular system of alloy. Roy et al.^13^ proposed an algorithmic framework using Generative Adversarial Networks (GANs) to generate novel compositions based on available data of MPEAs with higher hardness values. This is followed by identification of alloy with the highest hardness through a hardness predictor. While this approach enables discovery of new alloys, it is constrained to specific number of elements.

According to the literature, there is still a significant scope for research in the field of HEAs prediction. Previous studies have primarily focused on single-phase and multi-phase predictions, with limited exploration of other phases. Moreover, the development and validation of mechanical properties, such as hardness and yield, have largely relied on experimental approaches. However, there is a need to enhance the efficiency of ML models in predicting these properties by encompassing all possible scenarios. *A particular area that remains underdeveloped is the optimization of alloy composition with respect to mechanical properties*. This is mainly due to the vast compositional space of HEAs and the lack of nonlinear solving ML models that can effectively tackle this complexity. Therefore, the present study aims to address these gaps and develop ML models. We have successfully developed artificial neural networks that achieve an impressive Hamming accuracy score of 95% in predicting both single and multi-phases. This accuracy is reported to be the highest among previous studies focusing on multi-phase prediction. The primary objective of this research was to address the existing gaps in the literature by creating more accurate and generalized models for predicting the phases and mechanical properties of HEAs. Additionally, the authors developed a classifier to distinguish between Low and High Entropy Alloys, which can be further used for reduction in HEA search compositional space. Regressor models have been trained for predicting the hardness using alloy descriptors and predicted phase data. To advance HEA prediction, the study developed an algorithmic framework that optimizes the composition of HEAs to achieve higher hardness values.

## Results and discussions

### Alloy descriptors

In order to utilize alloys as input for machine learning models, it is crucial to transform their properties into mathematical representations. Previous studies have commonly employed compositional representations^14–16^, focusing on specific elements; we found out that it constrains the model to only process alloys made up using selected elements rather than generalizing across the entire compositional space of HEAs.

To address the issue of generality, this study employed a set of 29 thermodynamic, physical and semi-empirical properties as alloy descriptors to facilitate the identification, and prediction of phases and hardness in HEAs. Initial studies majorly relayed on thermodynamic properties including the entropy and enthalpy of equiatomic alloys for the phase stability. The mixing enthalpy and configurational entropy directly provide the relation for Gibb’s free energy as shown in Eq. (6). However higher configurational entropy stabilizes single phase solid solutions in the equiatomic alloy and vice versa with lower configurational entropies^17^. Similarly, Zhang et al.^18^ introduced a 3D graphical axis that incorporates ΔS_mix_, ΔH_mix_, and Delta to analyze different phases of MHEAs and bulk metallic glasses. In this study, the significance of ΔH_mix_ and δr phase descriptors, in addition to ΔS_mix_, was proposed for classifying various phases, including intermetallic, amorphous structures, and crystalline solid solutions. To enhance the prediction efficiency in different phases, studies started developing prediction models with physical and other descriptors. The rule of mixtures was used to transform basic material properties into these descriptors. These descriptors include average of atomic radius, melting point, Pauling electronegativity, Allen electronegativity, Valency Electron Concentration (VEC), itinerant electron per atom, atomic weight, density, molar heat capacity, and thermal conductivity. The basic rule of mixtures model equation is described in Eq. (1) in Table 1. Correspondingly, the standard deviation of the aforementioned descriptors was determined using Eq. (2). Shen Gue et al.^19^ demonstrated the importance of other parameters used in phase stability of HEAs apart from mixing entropy and proposed new parameters including electronegativity and VEC in this study for distinguish phases of FCC and BCC. Simultaneously, Zeng et al.^20^ utilized CALPAD and ML algorithms to derive multiple descriptors for phase prediction. Specifically, they derived Pauling electronegativity, Allen electronegativity, and VEC in their study. Later, Yang et al.^21^ developed the Ώ parameter for predicting solid-solution formation in MPEAs. The study developed the Eq. (8), and its boundary conditions of the Ώ for forming solid solution ability at MPHEA’s. Wang et al.^22^ introduced the ϒ parameter as a means to quantify lattice distortion, which differs from δr by comparing the average atomic size with the largest and smallest atoms among the constituents. In a similar manner, Singh derived the $\Lambda$ parameter as shown in Eq. (9) for understanding the differences between disordered solid solutions and compounds^23^. Efforts are underway to improve the efficiency of parameters usage in understanding the phase stability and its impact on final alloy properties. Similarly, this study conducted a vast parameter tunning operation for better phase and hardness predictions. Detailed analysis of the parameter correlation and its influence on mechanical properties were discussed in the subsequent sections.

**Table 1:** Mathematical equations and symbols of chosen alloy descriptors.

S.no.	Descriptor	Formula
1	Average values of elemental descriptors	$p= \sum_{i=1}^{n}XiPi$ X_i_ = elemental composition of ith element, Pi = elemental properties of ith element
2	Standard deviation	$\updelta = \sqrt{\sum Xi{(1-\frac{Pi}{P})}^{2}}$
3	Atomic volume^24^	$Vm= \sum_{i=1}^{n}Xi(\frac{4}{3}{\pi r}^{3})$
4	Mixing entropy^17^	$Smix= -R\sum_{i=1}^{n}Xi{\text{ln}}Xi$ R = 8.31* 10^–3^ kJ/Mol
5	Mixing enthalpy^25^	$Hmix=4\sum_{i=1 ,j\ne 1}^{n}\Delta Hij Xi Xj$
6	Gibbs free energy	$Gmix=Hmix-TavgSmix$
7	Average temperature^21^	$Tavg= \sum_{i=1}^{n}XiTmi$
8	Combination effect-1^21^	$\Omega = \frac{TavgSmix}{\left|Hmix\right|}$
9	Combination effect-2 ($\Lambda$)^23^	$\Lambda = \frac{Smix}{({\delta }_{r}{)}^{2}}$
10	ϒ^22^	$\Upsilon =(1-\frac{\sqrt{\frac{\left({\text{min}}\left\{ri\right\}+{r}^{2}\right)-{r}^{2}}{({\text{min}}\left\{ri\right\}+r{)}^{2}}}}{\sqrt{\frac{\left({\text{max}}\left\{ri\right\}+{r}^{2}\right)-{r}^{2}}{({\text{max}}\left\{ri\right\}+r{)}^{2}}}})$
11	Valency electron difference^20^	$\mathrm{\Delta VEC}= \sqrt{\sum_{i=1}^{n}Xi(VEC-VECi{)}^{2}}$

### HEAs identification

Most research studies in the field of HEAs modeling primarily focus on predicting phases or physical properties^4–12,14,16^. However, an inherent issue with these prediction models is that they are constructed based on the assumption that the input alloy will be a HEA. The input alloys may not necessarily conform to this constraint. Consequently, if a known non-HEA is provided as input, the resulting predictions of phases or physical properties will likely be incorrect, given the domain. Therefore, the identification of alloy is a crucial task preceding subsequent prediction.

Minh et al.^15^ developed a recommender system for generating new and identifying a given alloy as HEA based on the presence or absence of specific element combinations in the alloy. Their system is constrained by the allowable number of elements for alloy formation and focuses solely on equiatomic alloys, without considering different elemental concentrations for sets of elements. This highly restricts the exploration of new HEAs. Their system, when evaluated using a supervised learning approach, achieves an average accuracy of 80%.

**Figure 1:**
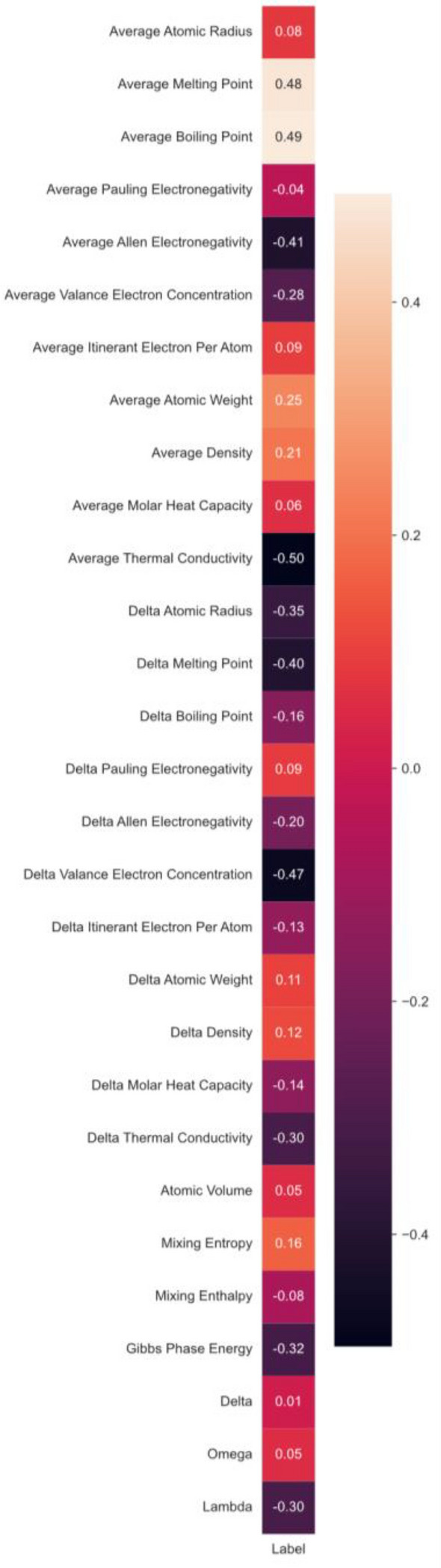
Pearson’s Correlation between alloy descriptors and High/Low entropy alloy label.

Utilizing the same datasets as Minh et al.^15^, we curated a comprehensive dataset comprising of 1156 alloys. This dataset comprises 471 and 685 equiatomic low entropy alloys (LEAs) and HEAs, respectively. We systematically eliminated all conflicts inherent in the dataset present within their study. We computed the above-specified 29 alloy descriptors for each equiatomic alloy. Now alloys are going to be represented in the form of 29-dimensional vector, irrespective of the elements used in the system of alloys. This will help us in the generalization of our classification model, as our model will be trained to recognize patterns in these dimensions, which rather than being affected by the presence or absence of an element will focus more on the concertation of constituent elements and their atomic properties. Pearson’s correlation coefficients were calculated for these alloy descriptors and their probability of being a HEA, to find the existence of any relation between them. In Fig. 1, we can see that Delta VEC, Average Thermal Conductivity, and Average Allen electronegativity show high negative, and average melting and boiling point show a high positive correlation with the probability of an alloy being HEA. This positive correlation attributes the ability of HEAs to withstand higher temperatures in the melting and boiling processes. HEAs are formed with higher interatomic bond strengths and phase stability, allowing them to withstand higher melting points^26^. Due to the various substitutional phases formed in the HEAs with varied temperature ranges, melting and boiling temperature parameters are positively correlated^26,27^. The decrease in the Delta VEC, average thermal conductivity, and average Allen electronegativity show higher possibilities of being HEA, which is in good agreement with the literature. Ji et al.^28^ demonstrated the influence of the electronegativity difference and delta (δ) values on the SS phases in the multi-principal alloys. The study provided a rule that lower electronegativity and δ are simple ways to demonstrate effective HEAs. Lower electronegativities compared to the consistent elements promote the solid solution, followed by a rise in mixing entropy^29^. Higher mixing entropy stabilizes the SS phases formed in the HEAs. Similarly, VEC and thermal conductivity are the most vital parameters in HEA formations^30,31^. Lowering the VEC promotes the disorderedness of the HEAs, allowing them to bond rather than be ordered. Additionally, VEC is simplistic and effective to use in HEAs.

**Figure 2:**
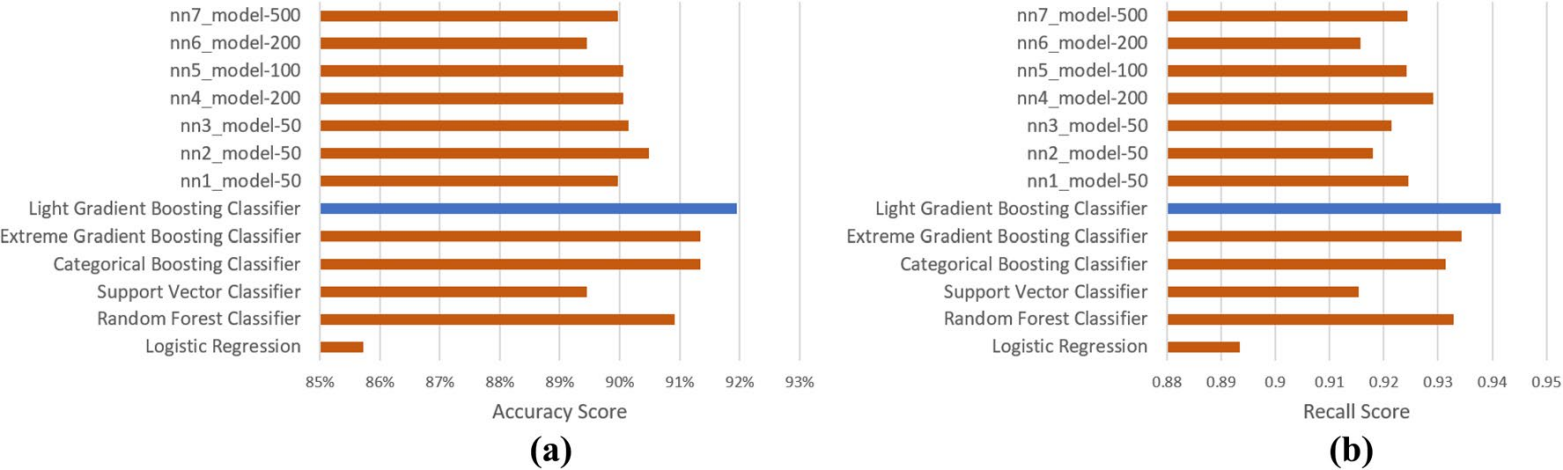
(**a**) Accuracy score and (**b**) Recall score of various models for identification of HEAs. The performance of the best model is highlighted in blue.

For the identification of HEAs, 13 different ML algorithms including gradient boosting algorithms and several architectures of neural networks have been tested. The performance of these models can be directly compared using accuracy and other metrics. However, our emphasis was directed toward the recall, specifying the ratio of true positives to the total number of actual positives. As HEAs being misclassified as LEAs could result in the rejection of alloys that might show desirable properties, a heightened recall score effectively reduces the likelihood of such instances occurring. We performed K-fold validation with 10 splits on each model so that we can better access their generalization capabilities. The outcomes of model testing are presented in Fig. 2, wherein the Light Gradient Boosting Machine^32^ (LightGBM) gave the highest accuracy and recall score of 91.9% and 0.94, respectively.

Despite exhibiting remarkable advancements compared to previous studies, this model is not exempt from limitations. Primarily, the dataset's confinement to equiatomic alloys imparts a constraint that potentially curtails the model's ability for generalized application, particularly when addressing alloys with diverse elemental compositions. Given that numerous datasets concerning phase or physical properties of HEAs predominantly encompass varied elemental compositions, this model's utility should not extend to compositional space reduction, as such an application might result in the undesired exclusion of promising alloys. Instead, its purpose should be aligned with furnishing researchers with a classification label regarding whether an alloy aligns with LEAs or HEAs, based on established literature.

### Multi-labeled phase classification

The mechanical and physical properties of HEAs exhibit variation according to their phase formations. Essential mechanical properties like hardness and tensile strength experience significant variations tied to distinct phase formations, making the need for phase prediction necessary. Different crystal structures, including FCC, BCC, HCP, and others, give rise to diverse properties contingent upon their respective arrangements. Furthermore, both single-phase and multiphase formations contribute to the alteration of mechanical properties. Therefore, there has been a surge in the pursuit of virtual alloy design, employing tools such as CALPHAD, ML, and AI. The prediction of phases and their correlation with mechanical properties serves to curtail the need for a large number of experimental trials, thereby fostering advancements in HEA applications. This approach accelerates HEA development by reducing empirical testing and facilitating its integration into real-world applications^1^.

**Figure 3:**
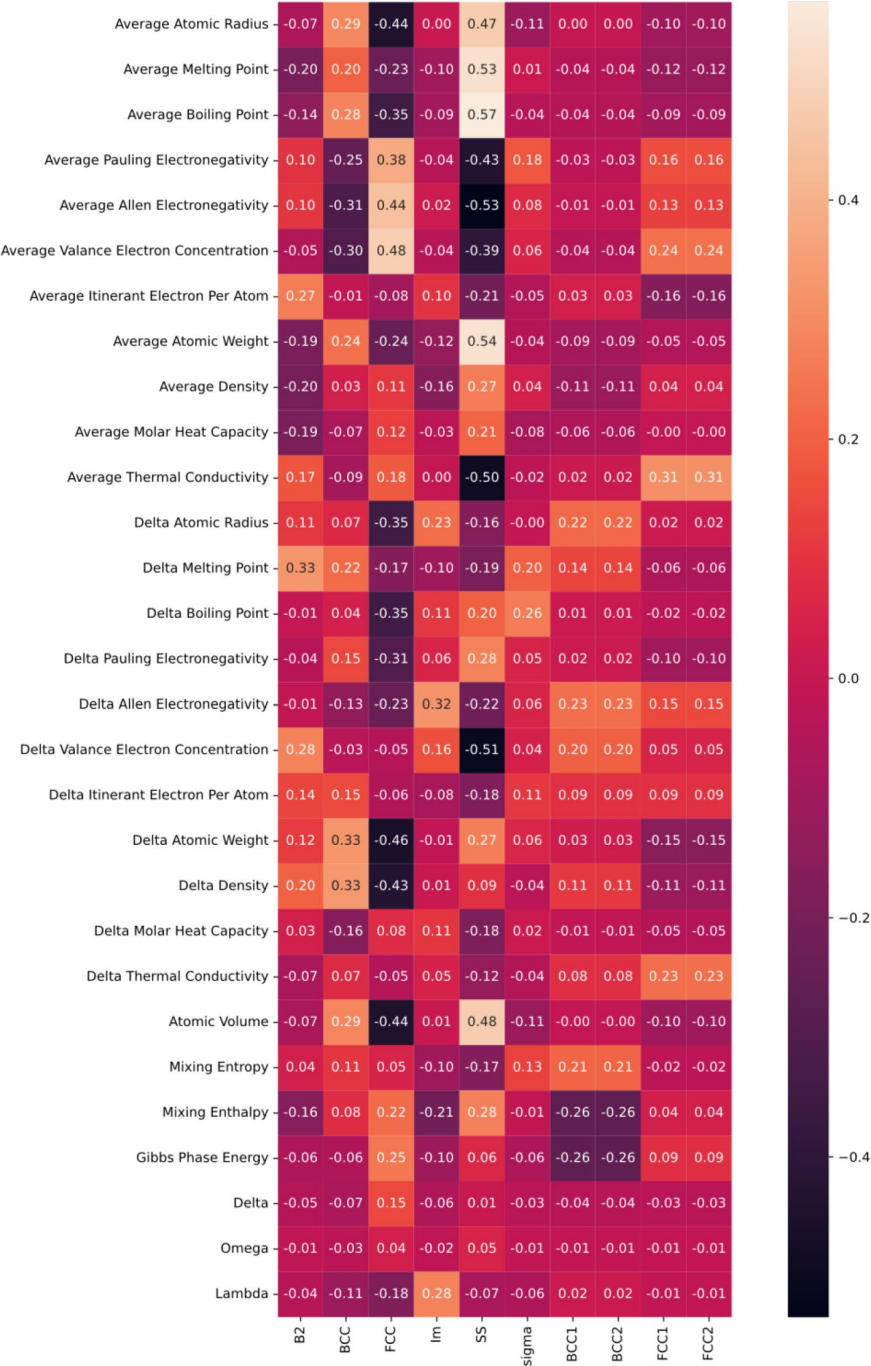
Pearson’s Correlation between alloy descriptors and predicted phases.

Multiclass classification limits the exploration of new inputs by constraining to a given set of single or multiphase classes, given the fact that HEAs have vast search compositional space of existence. Multi-label classification tries to bridge this gap by providing some degree of freedom for the possibility of the existence of a new combination of phases rather than being constrained to the predefined set of single and multi-phases. Shrey et al.^33^ included the processing route followed during the synthesis of alloy along with thermodynamic properties as an input. To achieve better results, we decided to increase the number of descriptors and expand the dataset used to train the model. A list of phase data for 1049 alloys along with their respective processing route has been compiled for this study. This dataset was further expanded up to 1184 to adjust for the bias for phases BCC1 + BCC2 and FCC1 + FCC2. The analysis of the relation between the alloy descriptors and the phases has been performed by computing Pearson’s correlation coefficient, presented in Fig. 3. Most of the properties show a positive or negative correlation with one or another phase, except Omega. Similar findings have been reported in previous studies^33^. However removing it didn’t have any positive effect on the performance of models during testing. Average atomic radius, average boiling point, and atomic volume showed a positive correlation with the BCC phase. These results are in good agreement with Shen Guo^34^. Similarly, the FCC phase has a higher correlation with average VEC, average Allen, and Pauling electronegativity^35^. Delta Allen electronegativity and Lamda showed positive influence on IM phase prediction and simultaneously, average atomic radius, boiling and melting points showed positive correlation in predicting the SS phase. Interestingly, Allen electronegativity showed a higher correlation with the greatest number of phases, followed by VEC^28–30^.

In this study, we developed a range of artificial neural network (ANN) models aimed at phase prediction. The primary distinction among these models lies in the number of layers and neurons within each layer. By training these models across varying epochs, we aimed to ascertain the potential occurrence of overfitting, thereby ensuring the robustness of our model selections. The final output layer of each model consists of 10 neurons, each representing a distinct phase, namely: B2, BCC, FCC, IM, SS, Sigma, BCC1, BCC2, FCC1, and FCC2. To interpret the output, we applied the Sigmoid activation function to obtain probabilities indicating the presence of a particular phase within the alloy.

**Figure 4:**
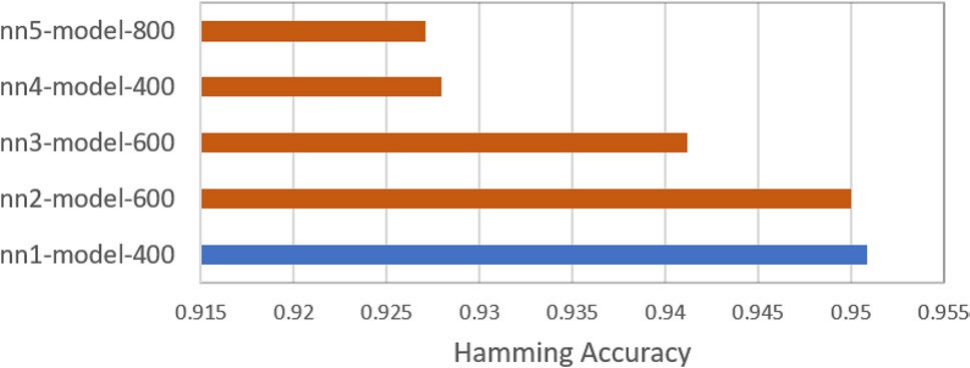
Hamming Accuracy Score achieved by various architectures of neural network models. The performance of the best model is highlighted.

The evaluation of models’ performance is conducted through K-fold cross-validation with 10 splits on each model and Hamming accuracy is calculated for the same. The comparative analysis of Hamming accuracy scores across various model architectures is present in Fig. 4. As models are trained at different epochs, this only includes the best results achieved by each model for all the epochs. Notably, the model designated as 'nn1-model-500' demonstrates the highest Hamming accuracy score of 95%.

### Hardness prediction

Previous studies on hardness prediction for HEAs have primarily concentrated on utilizing thermodynamic properties or elemental compositional representations. While these investigations have yielded remarkable predictive outcomes, they are often restricted to particular alloy types or a fixed number of elements. Jain et al.^36^ proposed an artificial neural network (ANN) model for the prediction of hardness of HEA. They were able to achieve a remarkable, R2 score of 0.95 but were limited to the prediction of said properties of alloys consisting of Al–Co–Cr–Fe–Mn–Nb–Ni−V. Consequently, the scope for exploring novel HEAs remains significantly limited.

**Figure 5:**
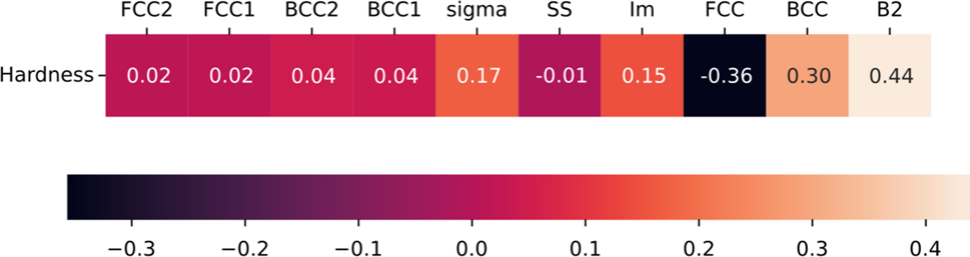
Pearson’s Correlation between predicted phase and Hardness (HV) values.

Pearson correlation analysis is conducted to assess the relationship between the predicted phases using our Multi-label Phase Prediction model taking “Arc Melting” as the processing route and the experimentally determined hardness values of 759 alloys, presented in Fig. 5. The B2 and BCC phases showed a higher positive correlation on hardness prediction. B2 phases are ordered phase formations in the HEAs with higher atomic bonding strength compared to other phases. Ordered structures allow restrictions on dislocations in HEA's, which allows them to enhance strain hardening. The authors have demonstrated the role of Al additions on B2 phase formation in subsequent sections with validation^37–39^. Similarly, the BCC phase showed a higher positive correlation with hardness because of its lower atomic packing density, solid solubility, and lower slip planes. Strain hardening of the HEA’s increases with a lower number of slip planes flowed by restricting the dislocation movement, which raises the hardness of the alloys. Simultaneously, the FCC phase showed a negative correlation with hardness properties. This can indicate that FCC has higher slip planes, causing it to deform rather than resist loads. Correspondingly, the Sigma and IM phases showed a moderate correlation with hardness as they formed with multiple elements with varying compositions and microstructures. Later phases, including the FCC1, FCC2, BCC1, and BCC2, showed minimal correlation with the hardness of the HEAs. This finding highlights the significance of the presence of phase in contributing to the alloy's mechanical properties. In addition to 29 alloy descriptors, we incorporated phase values as input for a set of regression models in our study.

**Figure 6:**
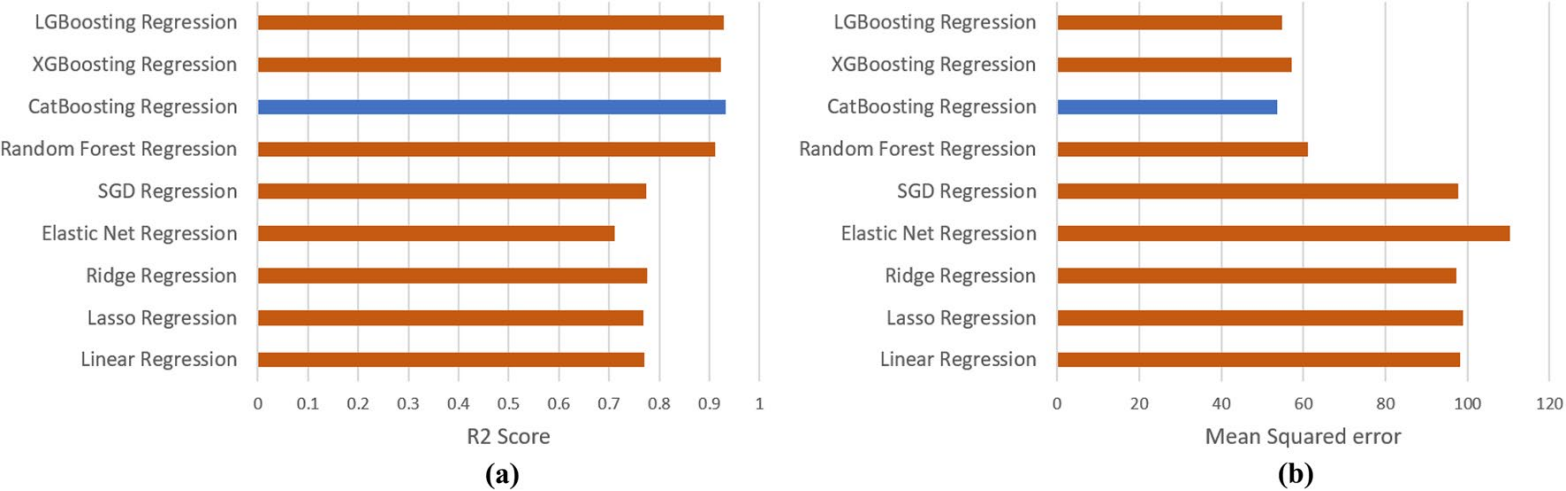
(**a**) R2 Score and (**b**) mean squared error of the models tested for hardness prediction. Performance of best model is highlighted.

In order to identify the optimal algorithm, nine machine learning algorithms were trained on the dataset, namely Linear Regression, Lasso Regression, Ridge Regression, Elastic Net Regression, SGD Regression, Random Forest, Categorical Boosting (CatBoosting), Extreme Gradient Boosting (XGBoosting), and Light Gradient Boosting Machine (LGBoosting). Performance evaluation was conducted using R2 score and root mean square error (RMSE), Fig. 6a,b, respectively. K-fold cross-validation with 10 splits was employed for calculating these metrics. Among the models, CatBoosting Regressor^40^ exhibited the highest R2 score of 0.933 and the lowest RMSE value of 53.12.

In addition to K-fold validation, the model exhibiting the highest R2 score undergoes validation using experimental data. The tabulated results in Table 2, provide a comprehensive overview, detailing the experimentally derived hardness (HV) values, the corresponding predicted values, and the associated percentage errors in the predictions.

**Table 2:** Validation of Hardness prediction model against experimentally calculated hardness values of HEA.

Alloys	Experimental hardness	Predicted hardness	Relative error (%)
Co10Cr20Fe30Ni40^41^	126.5	121.31	4.10
Al44.38Cr31.79Fe11.67Ni12.17^16^	771.27	779.71	− 1.09
Al1.2Cr17.42Fe25.42Ni28.32Ti27.62^16^	869.88	757.54	12.91
Al44.18Cr18.58Fe12.08Ni11.38V13.78^16^	898.18	790.52	11.99
Ti32Nb9Ta1Cr19Co39^16^	856	743.17	13.18
Al20Cr5Cu15Fe15Ni5Ti10V30^42^	650	693.37	− 6.67
Al32Co13Cr33Fe22^41^	735.5	730.24	0.72
Al41Co20Cr19Fe15Ni5^41^	762.8	814.63	− 6.79
Co6W9Al36Mo38Ni11^16^	725	659.06	9.10

Owing to the limited accessibility of experimental hardness data for HEAs, our model's capabilities remain constrained. The dataset comprises 17 elementals: Al, Co, Cr, Cu, Fe, Hf, Mn, Mo, Nb, Ni, Si, Sn, Ta, Ti, V, W, Zn, and Zr. Notably, a disproportional abundance of Al, Co, Cr, Cu, Fe, and Ni within the dataset could render our model particularly responsive to alloys incorporating these constituents. The predominant distribution of hardness values (HV) within the 300–800 HV range implies heightened predictive accuracy for alloys situated within this interval.

### Composition optimization

The exploration of HEAs poses a significant challenge due to their vast compositional space, making the search for alloys with desired properties computationally expensive. In particular, the optimization of hardness in HEAs presents additional difficulties. The predicted hardness landscape is characterized by non-continuity and non-differentiability at numerous points, further complicating the task of identifying optimal alloy compositions. Such complexities demand advanced computational approaches and algorithms capable of efficiently navigating the intricate landscape of hardness predictions in HEAs.

**Figure 7:**
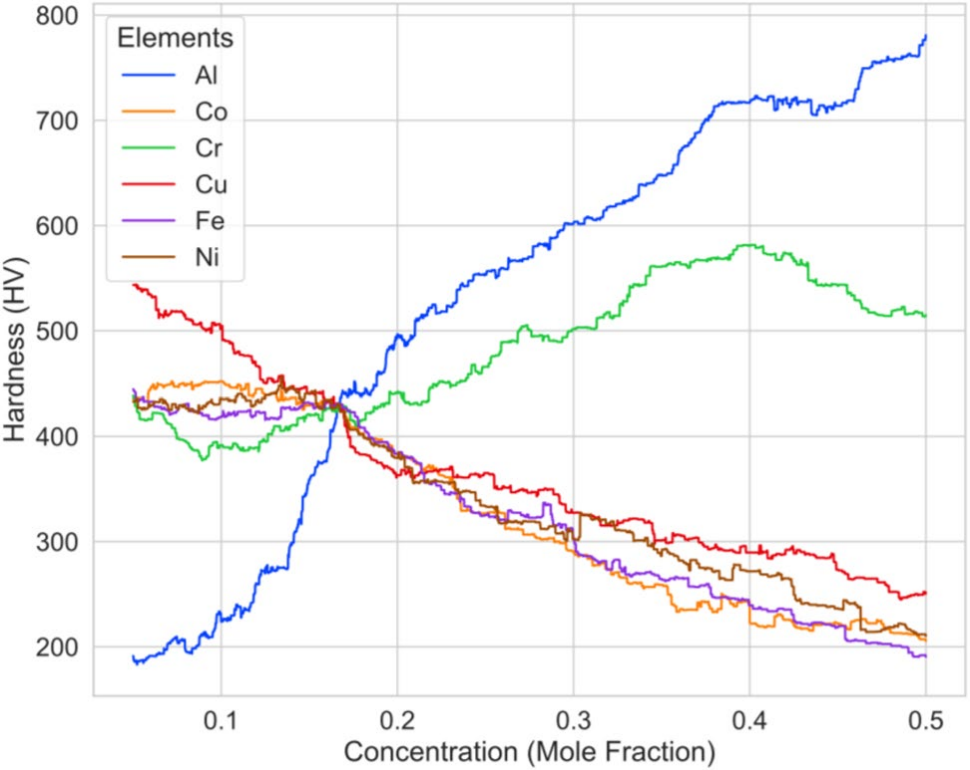
Hardness (HV) variation with the change in elemental concentration of the system of alloy AlCoCrCuFeNi.

We conducted the hardness analysis of a system of alloy consisting of 6 elements, AlCoCrCuFeNi. The concentration in terms of mole fraction of each element is varied incrementally by 0.0001 within the range of 0.05–0.5 taking one element at a time. The concentration of other elements is changed equiatomically to adjust for the incremental change in concentration of the element in question, to maintain a combined concentration of 1.0. This analysis serves as an illustrative demonstration, the concentration of elements apart from the selected element is altered in an equiatomic manner, thereby focusing solely on a restricted subset of potential elemental concentrations. The graph presented in Fig. 7, shows the change in hardness with the change of concentration. Each line in the graph shows an overall pattern of change in hardness values. The hardness of the alloy increases with the increase in the concentration of Al and Cr, and is vice-versa in the case of Co, Cu, Fe, and Ni. When observed locally, hardness changes erratically at many points and shows no pattern whatsoever.

**Figure 8:**
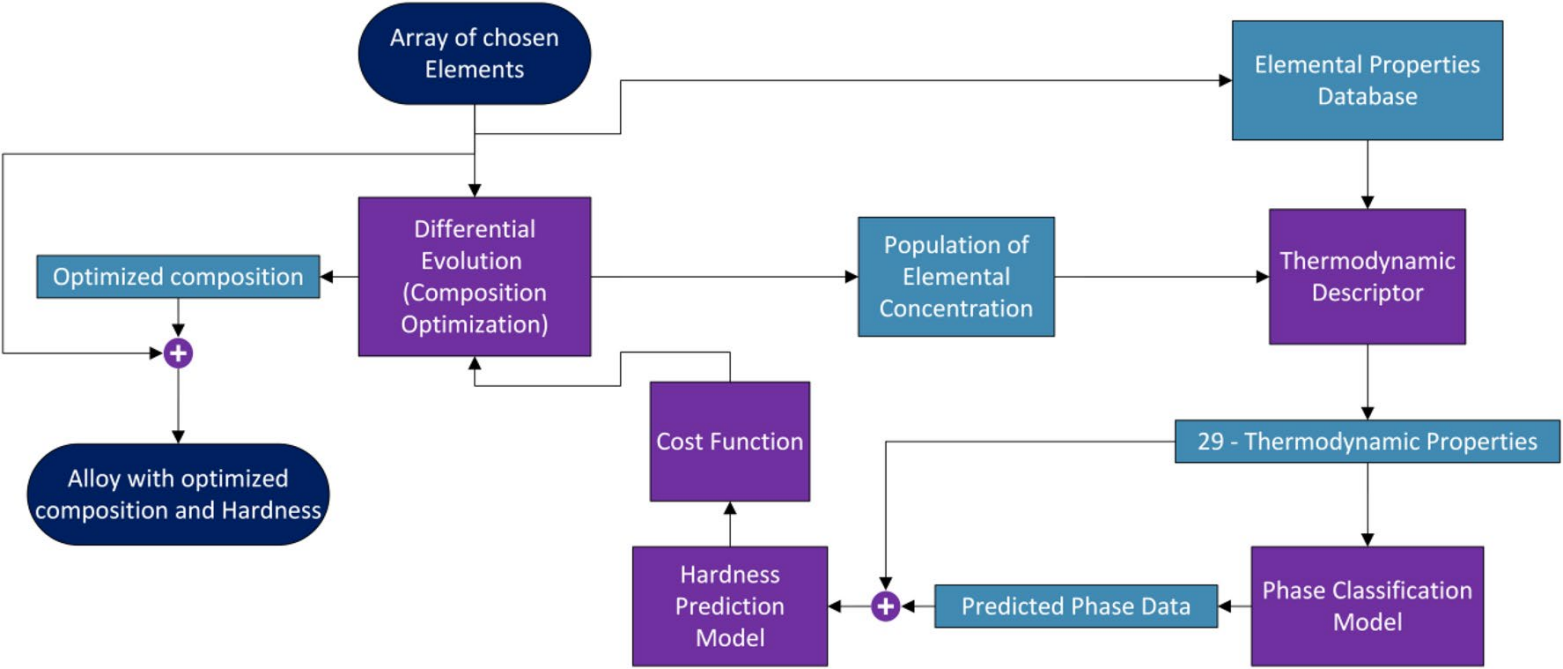
Flowchart of the proposed algorithmic framework for composition optimization for higher hardness values.

We present an algorithmic framework for optimizing the composition of alloys to achieve higher hardness values, targeting the objective of maximizing hardness while considering specified constraints. To accomplish this, we employ the Differential Evolution algorithm^43^ in our approach. To guide the algorithm effectively, we design a cost function that directs the evolutionary process towards the desired outcome. Additionally, we utilize a Multi-Labeled Phase prediction model to forecast the initial phase, which, along with alloy descriptors, aids in predicting the hardness of any given alloy. The flowchart presented in Fig. 8, shows a pictorial representation of the proposed algorithmic framework. However, we encounter a challenge: alterations in the concentration of one element, expressed as a mole fraction, should lead to corresponding changes in the concentration of other elements in different directions. Given the numerous potential combinations of how these changes affect other elements, maximizing hardness becomes a challenging search problem. To address this, we augment our cost function by penalizing deviations from the combined concentration of one element. This ensures that changes in one element's concentration result in proportionate adjustments in other elements, along with maximizing hardness.

**Figure 9:**
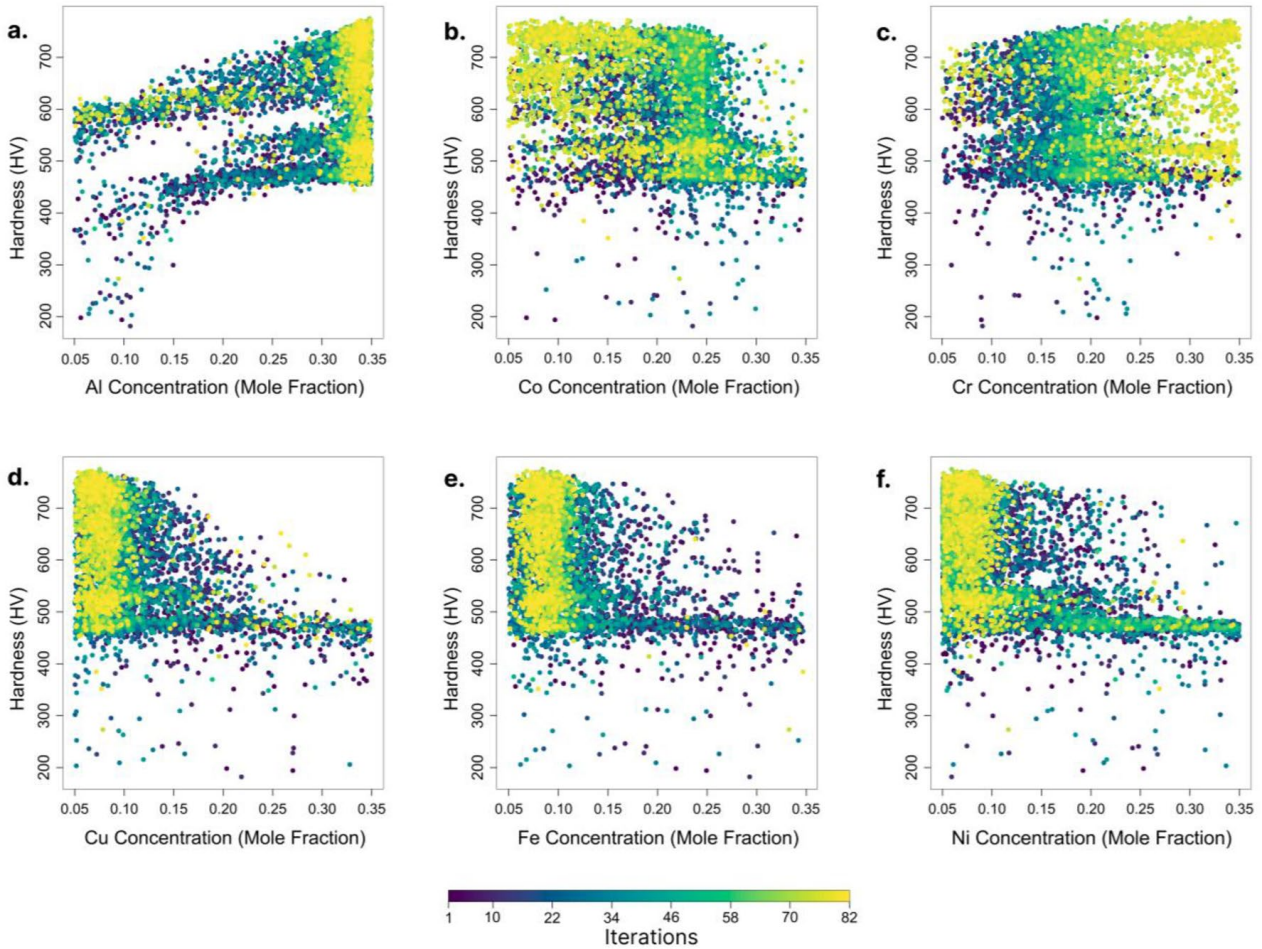
Hardness (HV) variation with change in concentration of (**a**) Al, (**b**) Co, (**c**) Cr, (**d**) Cu, (**e**) Fe, and (**f**) Ni during composition optimization of the system of alloy, AlCoCrCuFeNi.

To map out the path followed by the proposed algorithm during the optimization of the above-mentioned system of alloy, AlCoCrCuFeNi, we plotted all the concentration values considered for each element and the corresponding hardness values as the iteration progresses, in Fig. 9. At the initial stages, all the concentrations in the given limits of 0.05–0.35 are considered, as iteration progresses, points start to localize at a particular concentration space for all elements. For Al and Cr, at later stages of iteration, more of their higher elemental concentration values are considered in order to achieve better hardness. This is verified by the hardness analysis that we conducted for this system of alloy, Fig. 7, that the system of alloy tends to show higher hardness with the increase in the elemental concentration of Al and Cr. The same is true in the case of Co, Cu, Fe, and Ni. The algorithm favors lower elemental concentration for these elements as the hardness values decrease with the increase in their concentration.

Increasing the Al and Cr concentrations showed a hardness enhancement in the AlCoCrCuFeNi HEA because it promoted the formation of ordered B2 and disordered BCC phases in the alloy. The lattice mismatch between the B2 and BCC phases demonstrated the morphology of the following HEA, as shown in Fig. 9. Ma et al.^37^ investigated the formation of B2 and BCC phases in the AlxNiFeCoCr HEAs. The difference in the lattice mismatch in the BCC and B2 phases influenced the mechanical properties of the AlxNiFeCoCr^38^. Higher or lower lattice mismatches showed spherical or wavi-like morphology, and moderate lattice fits showed cuboidal particles with enhanced mechanical properties^39^. Similarly, increasing the Fe, Co, Cu, and Ni showed a drop in hardness, as shown in Figs. 7 and 9. Jain et al.^36^ confirmed a similar understanding with the feature importance and correlation on the hardness prediction. This investigation revealed that increasing the Fe, Cu, and Ni concentrations showed a negative correlation with the hardness of the AlCoCrFeNiVMoNb alloy.

We tested the proposed algorithm to optimize some systems of alloys. The system of alloys chosen for this purpose shows the least positive Relative Error in Table 2. The optimized concentration and the hardness value along with the predicted phase are mentioned in Table 3. The hardness values demonstrated are higher than the experimentally reported hardness value of the alloys.

**Table 3:** Demonstration of composition optimization algorithm.

Experimental alloy	Optimized alloy	Predicted phases (optimized alloy)
Al32Co13Cr33Fe22^41^ **(HV 735.5**)	Al34.08Co17.92Cr34.46Fe13.54 **(HV 748.85**)	B2 + BCC
Co10Cr20Fe30Ni40^41^ **(HV 126.5**)	Co22.86Cr34Fe34.99Ni8.15 **(HV 269.91**)	FCC
Co6W9Al36Mo38Ni11^16^ **(HV 725**)	Co19.87W16.13Al34.34Mo16.48Ni13.18 **(HV 731.41**)	B2 + FCC + σ

## Methods

### Data collection

To gather relevant data, this study reviewed a wide range of research articles and data analytics. For HEA identification purposes, the AFLOW^44^ and LTVC^45^ datasets, which were already classified as LEA and HEA, were considered. This data was then compiled into a dataset named dataset-1, consisting of 471 LEA and 685 HEA, conflicting data points were eliminated. After reviewing numerous papers, we focused on research that explored the phases formed by HEA along with the processing route taken to synthesize that alloy. Conflicting and duplicated data was excluded, resulting in a final dataset of 1049 phase data points, which is referred to as dataset-2^33,46^. This dataset includes 30 different phases such as B2, B2 + BCC, B2 + BCC + FCC, B2 + BCC + FCC + IM, B2 + BCC + FCC + Sigma, B2 + BCC + Sigma, B2 + FCC, B2 + FCC + Sigma, B2 + FCC1 + FCC2, B2 + Sigma, BCC, BCC + FCC, BCC + FCC + IM, BCC + FCC + Sigma, BCC + FCC1 + FCC2, BCC + IM, BCC_SS, BCC + Sigma, BCC1 + BCC2, BCC1 + BCC2 + FCC1 + FCC2, FCC, FCC + IM + Sigma, FCC_SS, FCC + Sigma, FCC1 + FCC2, IM, IM + BCC1 + BCC2, IM + FCC1 + FCC2, and Sigma + FCC1 + FCC2. This study obtained experimental hardness values for hardness prediction from the research papers, resulting in a collection of 759 hardness measurements of 17 elements^35,47,48^, referred to as dataset-3.

### Data pre-processing

The elemental compositions and elements are extracted from alloy representations in string form using regular expressions (Regex). To facilitate generalization, both molar ratio and percentage concentration representations are converted to mole fractions, indicating the respective element amounts in the alloy. Prior to deriving the alloy descriptors of the alloys, an atomic properties database is created, including attributes such as Atomic Radius, Melting Point, Boiling Point, Pauling Electronegativity, Allen Electronegativity, Valance Electron Concentration, Itinerant Electron Per Atom, Atomic Weight, Density, Molar Heat Capacity, and Thermal Conductivity. The computation of the descriptors is facilitated using vectorized matrix multiplication, after selecting the atomic properties from the database based on the constituent elements of the alloy. As each property exhibits highly variable ranges based on the underlying atomic property, all properties are standardized by subtracting the mean of the respective property after which they are divided by their standard deviation. This transformation promotes faster convergence during the learning phase of machine learning models. Within dataset-2, phases assume distinct classes, such as BCC + FCC or BCC + B2. These phase classes have been encoded as labels, facilitating their representation as a composite of 10 individual phases: B2, BCC, FCC, IM, SS, Sigma, BCC1, BCC2, FCC1, and FCC2. Therefore, each phase is now expressed as a 10-dimensional vector, fostering a more comprehensive and standardized representation.

### Neural network architecture for multi-labeled phase classification

The neural network model was constructed using the Keras library in Python. The model consisted of an input layer with 42 neurons, of which 29 neurons represent alloys descriptors and 13 neurons represent different routing processes involved in alloy synthesis, encoded as a one-hot vector. The model consists of only 1 fully connected hidden layer consisting of 64 neurons. It is followed by the Rectified Linear Unit Activation layer to introduce non-linearity and decrease the likelihood of vanishing gradient. Binary cross entropy is used as the loss function for training the model, so that each individual neuron in the last layer which represents the probability of the presence of a phase in the alloy, can be trained to function independently without being affected by the other neuron in the output layer. The model is trained for 450 epochs with Adam optimizer which, intuitively, is a combination of gradient descent with momentum algorithm and RMSProp optimization algorithm, for faster convergence.

### Hamming accuracy

The performance of the phase classification model is assessed in terms of Hamming accuracy. It considers the accuracy of the prediction of each label independently, i.e., the number of labels being predicted accurately. Mathematically, Eq. (1) represents the formula used to calculate Hamming accuracy.

1 $$\mathrm{Hamming\, Accuracy }= 1-\frac{1}{N\cdot L}\sum_{i=1}^{N}\sum_{j=1}^{L}XOR\left({y}_{ij},{\widehat{y}}_{ij}\right)$$

The output layer of the phase classification model produces output within the (0, 1) interval, attributed to the utilization of a Sigmoid activation function. These values are converted into binary form by way of thresholding. The threshold value is calculated using the trial-and-error method. A function iterates through all possible values of threshold up to a precision of 3 decimal places, and the chosen threshold corresponds to the point yielding the minimum Hamming score, indicative of the least labels incorrectly classified. This iterative procedure is replicated for each model subsequent to training.

### Differential evolution

Differential evolution^43^ (DE), a metaheuristics algorithm employed for optimizing the composition of HEAs, has been implemented in our approach using the SciPy^49^ package. Metaheuristic algorithms are able to obtain an optimal solution for very complex problems like HEA compositional search space^50^. They offer satisfactory outcomes with reduced computational demands when compared to exhaustive iterative procedures that involves testing all conceivable elemental compositions. DE initially generates a set of compositions, which expands proportionally with the number of elements, serving as the initial candidate solutions. Subsequently, these candidates undergo mutation and crossover operations with the existing set to generate a new set of candidates. Should the solutions of the new candidates surpass the existing ones, they replace the latter based on the evaluation of a designated cost function. We conducted an evaluation of various hyperparameters, including optimization strategies and mutation-recombination constants. This exploration aimed at identifying optimal parameter configurations that facilitate enhanced exploration and faster convergence within the compositional space of HEAs for hardness optimization. The default population size and recombination constant, i.e., 15 times the number of elements in the alloys, and 0.7 performs best, respectively. The mutation constant is randomly changed between the range (0.0, 1.0), and ‘Sobol’ method is used to generate the initial population for DE. “best2bin” strategy is used to improve the candidate solutions, and tolerance is chosen to be 0.01 which terminated the optimization when the standard deviation of the population falls below a certain limit. We have implemented the algorithm in a way to exploits the vectorization capabilities of DE. This allows for faster computation, as the cost for the whole population is predicted in a parallelized manner (Supplementary Information).

### Cost function

The cost function which is at the core of composition optimization for higher hardness, consists of two essential components. In mathematical form, the cost function can be represented by the formula given in Eq. (2).

2 $${\text{Cost}}\;\;{\text{Function}} = - \left( {{\text{PH}}{-}\alpha *{\text{CD}}} \right)$$

Here “PH” is the predicted hardness (HV) value for an alloy in question. “CD” represents the amount of deviation in the elemental concentration of the alloy from the total of 1.0, i.e., if the sum of the concentration of all the elements is equal to 0.97 then the value of “CD” is going to be 0.03. The parameter alpha, a constant multiplier, typically assumes a higher value to prioritize the sum total of elemental concentrations equating to 1.0. This strategic selection is crucial to prevent the algorithm from generating outcomes that are impractical or unforeseen. At a relatively lower alpha value, the algorithm can attain high hardness values; however, it might deviate from the concentration constraint, possibly yielding an alloy with substantially heightened hardness but impractical elemental concentrations. Conversely, opting for a relatively higher alpha value ensures that the concentration constraint is followed but will limit the algorithm’s capabilities of exploration.

### Supplementary Information

Supplementary Information.

## Data Availability

The codebase associated with the reported work is available at: https://github.com/anshpoonia/Design_of_HEAs
